# Myochrysine Solution Structure and Reactivity

**DOI:** 10.1155/MBD.1994.363

**Published:** 1994

**Authors:** R. C. Elder, William B. Jones, Zheng Zhao, John G. Dorsey, Katherine Tepperman

**Affiliations:** 1 Biomedical Chemistry Research Center Department of Chemistry and Biological Sciences University of Cincinnati Cincinnati OH 45221-0172 USA; 2 Mallinckrodt Medical Inc. 675 McDonnell Blvd P.O. Box. 5840 St. Louis MO 63134 USA; 3 Merck and Co. Inc. WP 78-110 West Point PA 19486 USA; 4 Chemistry Department Florida State University Tallahassee FL 32306 USA

## Abstract

We have determined the framework structure of Myochrysine (disodium gold(I)thiomalate) in the solid state and extremely concentrated aqueous solution, previously. It consists of an open chain polymer with linear gold coordination to two thiolates from the thiomalic acid moieties which bridge between pairs of gold atoms providing an Au-S-Au angle of 95°. The question remained: was this structure relevant to the dilute solutions of drugs administered and the still lower concentrations of gold found in the bodies of patients (typically 1 ppm Au in blood and urine or 5 μM in Au). We have provided an answer to that question using extended X-ray absorption spectroscopy (EXAFS) and capillary zone electrophoresis (CZE). EXAFS studies confirm that the polymeric structure with two sulfur atoms per gold atom persists from molar concentrations down to millimolar concentrations. CZE is able to separate and detect Myochrysine at millimolar levels. More importantly, at micromolar levels Myochrysine solutions exhibit identical CZE behavior to that measured at millimolar levels. Thus, aqueous solutions of the drug remain oligomeric at concentrations commensurate with those found in patient blood and urine.

The reactivity of Myochrysine with cyanide, a species especially prevalent in smoking patients, was explored using CZE. Cyanide freely replaces thiomalic acid to form [Au(CN)_2_]^-^ and thiomalic acid via a mixed ligand intermediate. The overall apparent equilibrium constant (K_app_) for the reaction is 6×10^-4^M^-1^. Further reaction of [Au(CN)_2_]^-^ with a large excess of L, where L is cysteine, N-acetylcysteine, or glutathione, shows that these amino acids readily replace cyanide to form [AuL_2_]^-^. These species are thus potential metabolites and could possibly be active forms of gold in vivo. That all of these species are readily separated and quantified using CZE demonstrates that capillary electrophoresis is an accessible and powerful tool to add to those used for the study of gold-based antiarthritis drugs.


### MYOCHRYSINE SOLUTION STRUCTURE AND REACTIVITY

R.C. Elder*, William B. Jones@, Zheng Zhao#,
John G. Dorsey+ and Katherine Tepperman

Biomedical Chemistry Research Center, Department of Chemistry and Biological Sciences,
University of Cincinnati, Cincinnati, OH 45221-0172, USA

### Abstract

We have determined the framework structure of Myochrysine (disodium gold(I)thiomalate) in the solid state and extremely concentrated aqueous solution, previously. It consists of an open chain polymer with linear gold coordination to two thiolates from the thiomalic acid moieties which bridge between pairs of gold atoms providing an Au-S-Au angle of 95°. The question remained: was this structure relevant to the dilute solutions of drugs administered and the still lower concentrations of gold found in the bodies of patients (typically 1 ppm Au in blood and urine or 5 µM in Au). We have provided an answer to that question using extended X-ray absorption spectroscopy (EXAFS) and capillary zone electrophoresis (CZE). EXAFS studies confirm that the polymeric structure with two sulfur atoms per gold atom persists from molar concentrations down to millimolar concentrations. CZE is able to separate and detect Myochrysine at millimolar levels. More importantly, at micromolar levels Myochrysine solutions exhibit identical CZE behavior to that measured at millimolar levels. Thus, aqueous solutions of the drug remain oligomeric at concentrations commensurate with those found in patient blood and urine.

The reactivity of Myochrysine with cyanide, a species especially prevalent in smoking patients, was explored using CZE. Cyanide freely replaces thiomalic acid to form $[Au(CN)_2]^-$ and thiomalic acid via a mixed ligand intermediate. The overall apparent equilibrium constant ($K_{app}$) for the reaction is $6 \times 10^{-4}$ $M^{-1}$. Further reaction of $[Au(CN)_2]^-$ with a large excess of L, where L is cysteine, N-acetylcysteine, or glutathione, shows that these amino acids readily replace cyanide to form $[AuL_2]^-$. These species are thus potential metabolites and could possibly be active forms of gold in vivo. That all of these species are readily separated and quantified using CZE demonstrates that capillary electrophoresis is an accessible and powerful tool to add to those used for the study of gold-based antiarthritis drugs.

### Introduction

The gold-based drugs used in rheumatoid arthritis therapy are extraordinary in that they have been prescribed for 70 years or more while their mode of action is not yet understood. Also these drugs are extremely labile, converting to a variety of biotransformation products once they are administered. Thus, the active agent is unknown as well.

In this paper we attempt to deal with some of this uncertainty. We have previously examined the structure of disodium gold(I)thiomalate (Myochrysine, AuTM) and found that it is oligomeric with sulfur bridges between gold atoms forming a helical structure with an oligomerization number of approximately six (1). The gold-sulfur framework structure is shown in Figure 1. This open chain structure is end capped

---

@ Current address: Mallinckrodt Medical Inc., 675 McDonnell Blvd., P.O. Box 5840, St. Louis MO 63134
### Current address: Merck and Co. Inc., WP 78-110, West Point PA 19486
+ Current address: Chemistry Department, Florida State University, Tallahassee, FL 32306

by a seventh thiomalate ligand giving a ratio of Au:S of 6:7 (2).

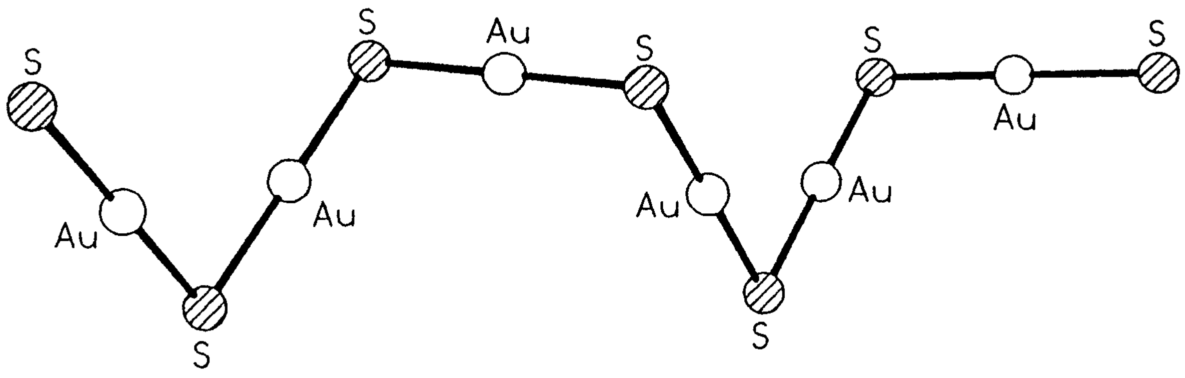

FIGURE 1: PROPOSED STRUCTURE OF MYOCHRYSINE. ONLY THE GOLD AND SULFUR ATOMS ARE DISPLAYED FOR SIMPLICITY. THE SULFUR ATOMS ARE BOUND TO GOLD AND FORM A 180 DEGREE ANGLE AND THE GOLD–SULFUR–GOLD ANGLE IS 95 DEGREES. THERE IS A 90 DEGREE TORSIONAL ANGLE ABOUT THE SULFUR–GOLD–SULFUR LINKAGE THAT ALLOWS FOR THE CLOSE $AU_1$–$AU_3$ CONTACT SEEN IN THE WAXS/DAS DATA.

We also had received from Drs. Helen Howard Lock and Colin Lock of McMaster University samples of optically pure sodium gold(I) thiomalate. That is, samples synthesized using either the D- or L-form of thiomalic acid as opposed to the enantiomeric mixture of the acids normally used in the synthesis of AuTM. We wished to determine whether there were any differences between the optically pure forms and the enantiomeric mixture which could be determined by EXAFS studies.

Also, we were concerned whether the drug structure, determined either with the solid drug or in extremely concentrated solution, had any bearing on the drug as it exists after administration at typical concentrations of a few parts per million gold (micromolar concentrations) in the blood stream. To determine whether the same structure persists at such low concentrations we have utilized a two part strategy. First, we have examined the structure using extended X-ray absorption fine structure (EXAFS) at millimolar concentrations. Second, we have characterized the drug using capillary zone electrophoresis at millimolar levels and also at the micromolar levels of gold which are found in patients' blood. Details of those studies are presented here.

Since we have recently found dicyanogold(I) circulating in the blood and excreted in the urine of patients receiving various gold drugs (3), we wished to see both how that complex might form and how reactive it is, especially toward some of the thiols such as cysteine and glutathione which are naturally available in the body. These reactions have been characterized using capillary zone electrophoresis as well and the results presented here.

### Materials and Methods

**Reagents and Materials.** Myochrysine (disodium gold(I) thiomalate) and thiomalic acid were purchased from Aldrich Chemical Co. (Milwaukee, WI), Potassium dicyanogold(I) was purchased from Sigma (St. Louis, MO) and Potassium cyanide was purchased from Mallinckrodt Chemical Co.(St. Louis, MO). D and L forms of Myochrysine were a gift from Drs. Colin and Helen Howard Lock of McMaster University. All reagents were used without further purification except where noted.

**Extended X-ray Absorption Fine Structure (EXAFS).** Solid samples were prepared by diluting 10 mg of each sample with 60 mg of finely ground boron nitride and placed in a cell with a 1 mm path length and cross-section of 3 by 27 mm. Solution samples were made by dissolving the requisite mass of myochrysine in 10 mL of water to make samples that were 3.06 and 5.03 mM. Approximately 0.1 mL of each solution was loaded into separate cells with a 1 mm path length.

EXAFS data for solids were collected at SSRL beamline IV-3, whereas those for solutions were measured at NSLS beamline X19A. Data collection methods were essentially the same for both beamlines with the exception that the solution data were measured at room temperature and the solids at 10 K. Transmitted and incident intensity were measured using standard nitrogen-filled ionization chambers. Simultaneous calibration of the wavelength was performed by passing the transmitted intensity through a gold foil standard. Fluorescence data were collected concurrently using a Stern, Heald detector fitted with a germanium filter to stop scattered radiation. Data were collected from 300 eV below the Au-$L_{III}$ edge to 1040 eV above the Au-$L_{III}$ edge. A locally modified version of XFPAK (Robert Scott, University of Georgia) was used to process the data.

**Capillary Zone Electrophoresis.** A Beckman P/ACE 2050 (Beckman, CA) with an on-line UV detector at 214 nm was used for the electrophoresis studies. Pressure injections were used to introduce the sample onto a 75 micrometer I.D. fused-silica capillary column (Polymicro Technologies, Phoenix, AZ). The total length of the column is ca. 20 cm from injection point to detection point. Each new capillary was pretreated with 0.1 M phosphoric acid for two hours and then flushed thoroughly with water, 20 mM sodium hydroxide and finally the buffer solution. Prior to each injection the capillary was rinsed with 10 mM sodium hydroxide and buffer solution. The capillaries were checked daily against a standard for reproducibility. Joule heating was compensated for by cooling the capillary with a circulating coolant jacket which maintained the temperature at 30.00+0.01°C.

### Results

**EXAFS of Solid and Solution Myochrysine.** The pseudo radial distribution functions calculated from the three solid samples: L, D, and racemic forms of AuTM are shown in Figure 2 and show no recognizable differences from sample to sample. The principal peak near 1.9 Å was back transformed in each case and fit to model parameters taken from a solid sample of $Na_3[Au(S_2O_3)_2]$. The results were as follows: D-AuTM: n=2.0, d=2.29 Å, DE=2.0 eV, sigma=0.052 Å and Fit=0.20; L-AuTM: n=2.1, d=2.29 Å, DE=1.7 eV, sigma=0.056 Å and Fit=0.14; racemic-AuTM: n=1.8, d=2.30 Å, DE=0.3 eV, sigma=0.048 Å and Fit=0.19, where n is the coordination number, d is the bond distance, DE is the shift in $E_0$ (the onset energy for EXAFS), sigma is the disorder parameter and Fit is the goodness of fit to the model.

Solution data were measured for D-AuTM on a 3.06 mM solution and for L-AuTM on a 5.03 mM solution. Previously we had measured a racemic-AuTM solution of 15.0 mM (1). Pseudo radial distribution functions for 3.06, 5.03 and 15.0 mM aqueous solutions of D-, Land racemic-AuTM respectively contain a single intense peak, indistinguishable from those shown in Figure 2. The peaks were transformed into k space and modeled with empirical parameters extracted from the solid sample of $Na_3[Au(S_20_3)_2]$. Coordination numbers calculated for the dilute solutions (2.1 atoms for 3 mM D Myochrysine and 2.1 atoms for 5 mM L Myochrysine) are in excellent agreement for those calculated for the corresponding solid samples (2.0 and 2.1 atoms respectively). Although coordination numbers from EXAFS have large errors associated, these values are well within the generally accepted limits for a two coordinate species.

Solution bond distances are 2.31 A for both the D and L forms and 2.30 A for the racemic form (4). Differences of 0.01 A are statistically insignificant for bond distance calculations and therefore the distances calculated for all of the above samples are considered the same.

**CZE of Low Concentration Aqueous Solutions of Myochrysine.** Capillary zone electrophoresis is a conceptually simple experiment in which a buffer-filled, open silica capillary column is placed between two electrolyte reservoirs as indicated in Figure 3. Sample is introduced into the capillary by hydrostatic pressure or electromigration. After the capillary is placed in the electrolyte a potential difference is applied across the capillary. Separation of molecules and ions using CZE is based on their charge densities. Conventional CZE operates with the detector near the cathode and the sample is injected at the anodic end. In this arrangement cations are moved rapidly toward the detector by electrophoresis while neutral and anionic species migrate more slowly based on electroosmotic flow (EOF). Subtle differences in the charge or volume of the molecule will change the electromigration time (5). Thus a change in composition of Myochrysine as a function of concentration will result in a shifting of the corresponding peak in the electropherogram. Electropherograms of AuTM solutions are shown in Figure 4. Individual components may be identified by their retention times or with a diode array spectrometer as detector by their spectra. For the electropherogram shown in Figure 4, the separation between the injection point and the variable wavelength detector operated at 214 nm was 20 cm . A 20 mM phosphate buffer at pH 8.0 was used with a potential difference of 8 kV.

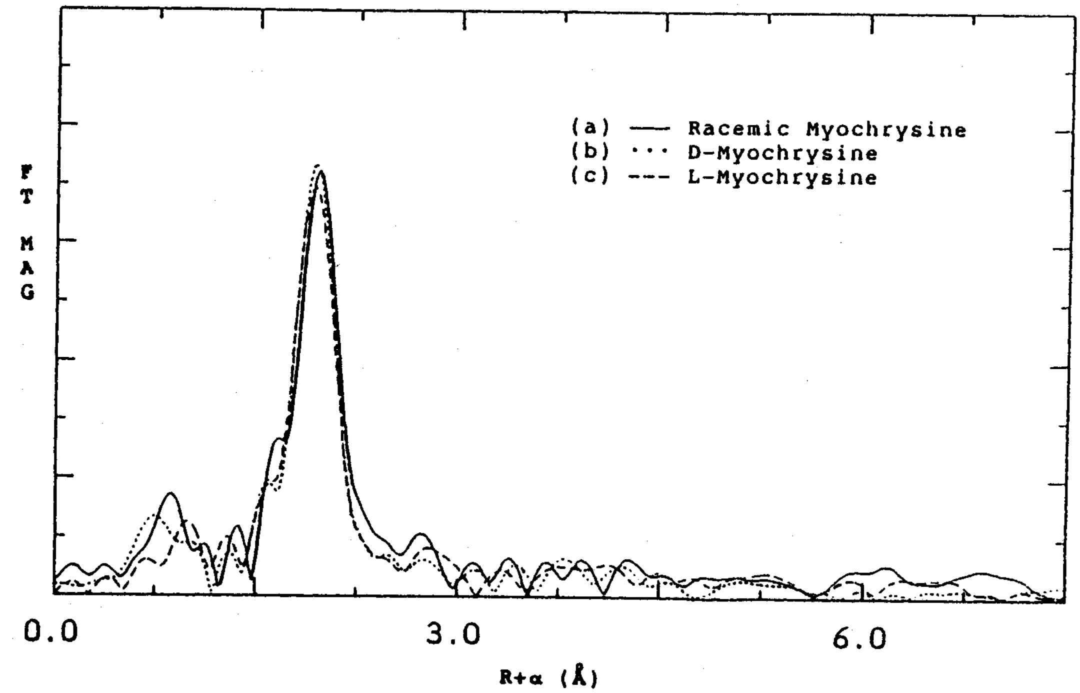

FIGURE 2 COMPARISON OF THE PSEUDO RADIAL DISTRIBUTION FUNCTIONS FORTHE D, L, AND RACEMIC FORMS OF MYOCHRYSINE IN THE SOLID PHASE. THE PEAK SHOWN IS THE GOLD–SULFUR PEAK. NO PEAK CORRESPONDING TO A GOLD–GOLD INTERACTION IS PRESENT. THE PRDF'S ARE VIRTUALLY IDENTICAL AND SHOW NO DIFFERENCES IN STRUCTURE.

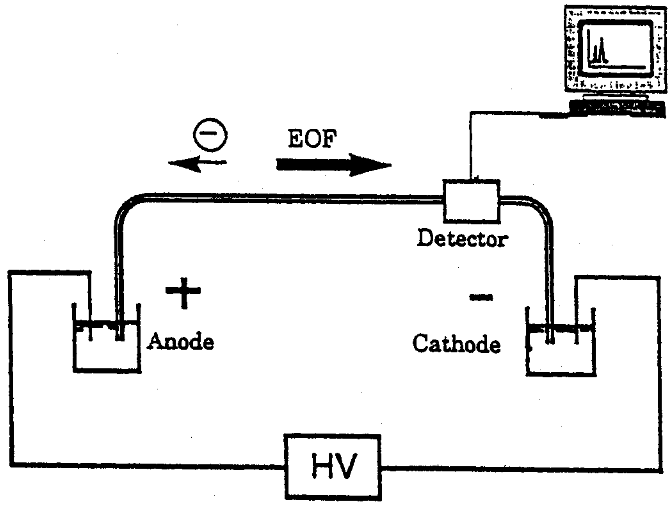

FIGURE 3. SCHEMATIC DIAGRAM OF CAPILLARY ELECTROPHORESIS INSTRUMENT.

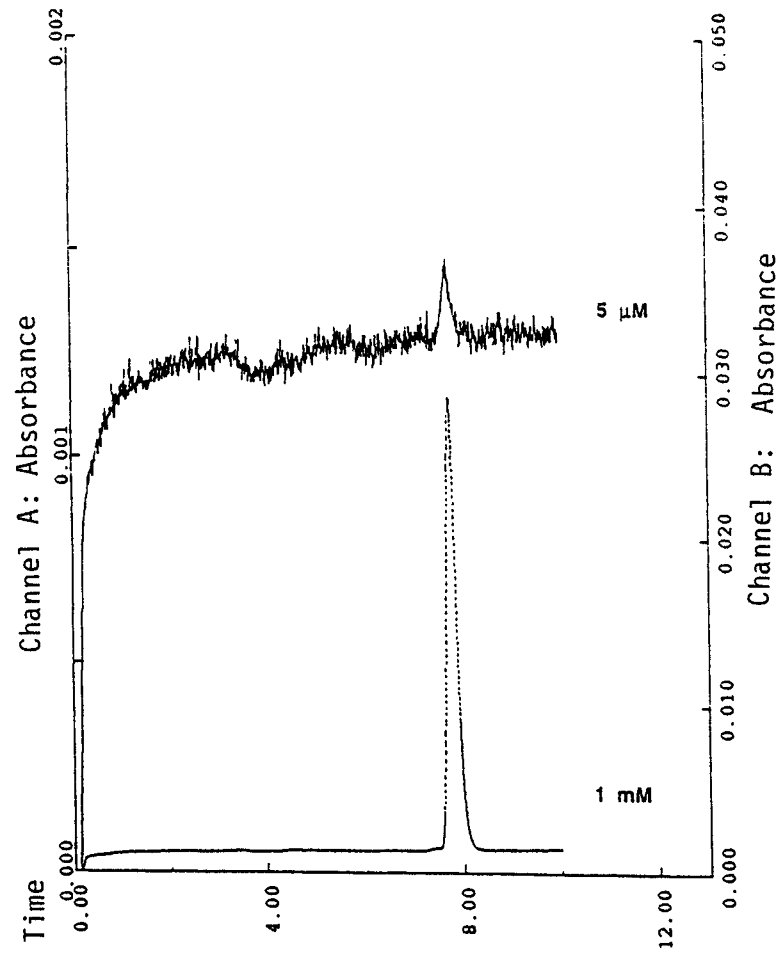

FIGURE 4. COMPARISON OF THE ELECTROPHEROGRAMS OF (UPPER TRACE) 5µM AND (BOTTOM TRACE) 1 MM MYOCHRYSINE IN 20 MM PHOSPHATE BUFFER, PH 8.

Myochrysine was dissolved at various concentrations ranging from millimolar as was used for the EXAFS studies to the micromolar gold concentrations found in patient's blood and urine. The solvent was 20 mM sodium phosphate buffer adjusted to pH=8.0 using phosphoric acid and sodium hydroxide. With careful control of column pretreatment and rinsing, migration times are highly reproducible. Thus for 10 samples of AuTM, the average migration time was 8.61 min with a standard deviation of 0.14 min or a relative standard deviation of 1.6%. Peak shape and electromigration time in the electropherograms are identical at both millimolar and micromolar concentrations of AuTM (see Figure 4).

**Solution Studies of Myochrysine and Cyanide.** Our previous studies of patients undergoing AuTM therapy show that dicyanogold(I) is a major metabolite, accounting for as much as 30% of the excreted gold (3) which appears in urine. Some free dicyanogold(I) is found in the blood as well. Cyanide is inhaled from cigarette smoke in the form of hydrocyanic acid and also occurs naturally as a product of the oxidative burst of polymorphonuclear leukocytes (6). Once injected into a patient, AuTM can undergo ligand exchange reactions with free cyanide which may hinder, modify or enhance its therapeutic properties.

Graham et al. followed the reaction of potassium cyanide and Myochrysine using $^{13}C$ and $^{1}H$ NMR techniques. They concluded that dicyanogold(I) was formed via a mixed ligand intermediate which maximized at a 1:1 molar ratio of cyanide to Myochrysine. Because of severe line broadening and overlap of signals at a ratio higher than 2:1 cyanide to Myochrysine they were not able to study larger ratios. Due to the relative insensitivity of NMR, they were unable to follow reactions at physiological levels and instead used concentrations at millimolar levels (20 mM for C NMR). We were able to follow the same reaction at a ratio up to 10:1 cyanide to Myochrysine using CZE with UV detection at 214 nm and determine an overall equilibrium constant.

Initially myochrysine, thiomalic acid and dicyanogold(I) were individually separated using CZE to determine the optimum conditions for elution. Applied voltage, pH and reproducibility were systematically studied to determine the best conditions for separation. Linear calibration curves of absorbance vs. log concentration were calculated for use in determining the equilibrium constants.

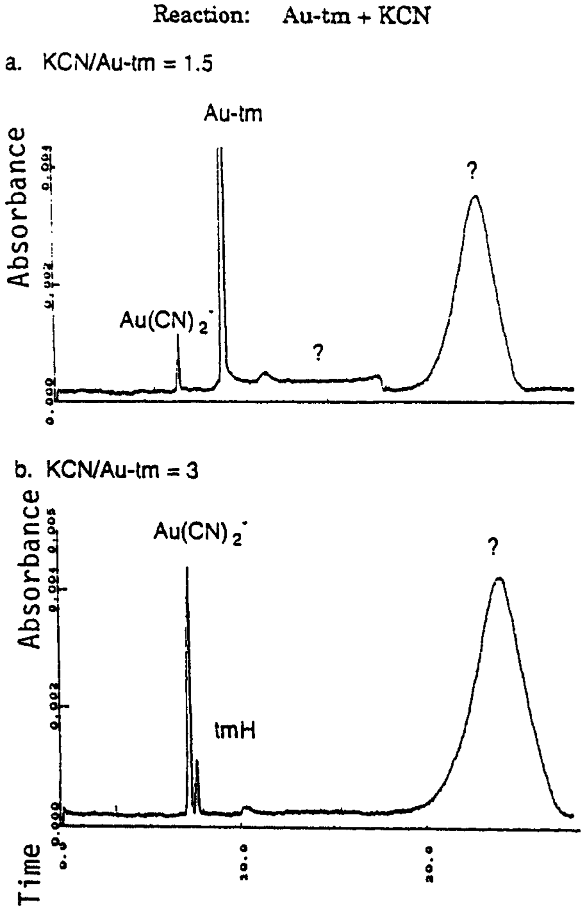

FIGURE 5 ELECTROPHEROGRAMS OF REACTION MIXTURE OF AuTM WITH KCN 27 CM CAPILLARY; 8 KV; 214 NM; 20 MM PHOSPHATE, PH 8.0

Myochrysine was dissolved in 20 mM aqueous phosphate buffer to make a 1.0 mM solution. The PH was adjusted to 8.0 with phosphoric acid and sodium hydroxide. Aliquots were taken at the initial concentration and at regular intervals as the solution was titrated with KCN. After the first addition of KCN three peaks in the electropherogram corresponding to AuTM, dicyanogold(I) and a mixed ligand species were separated (see Figure 5). As the concentration of cyanide was increased to 2:1 cyanide to AuTM the concentrations of mixed ligand species and dicyanogold( I ) increased and the gold thiomalate decreased. Additional KCN caused a continued decrease in the concentration of AuTM and the mixed ligand species also began to decrease. AuTM was completely converted to the mixed ligand species and dicyanogold(I) at a ratio of 3:1 KCN to AuTM. Dicyanogold(I) was the only species left in solution after a ratio of 4:1

cyanide to Myochrysine was reached. These results, presented in Figure 6, clearly show that AuTM is converted to dicyanogold( I ) via a mixed ligand intermediate supporting the earlier NMR work done by Graham. The small discrepancy in the maximum formation of the mixed ligand intermediate may be attributable to the difference in buffer concentrations used for the CZE and NMR experiments. High buffer concentrations in CZE cause high voltage leakage problems thereby forcing us to use a much lower buffer concentration than used in the NMR experiments.

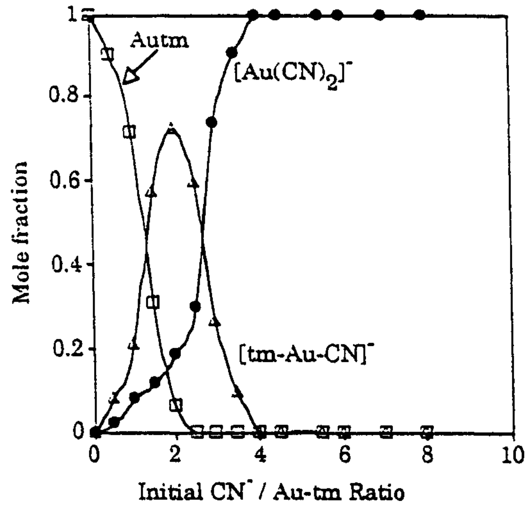

FIGURE 6 TITRATION OF AUTM WITH KCN. 27 CM FUSED-SILICA CAPILLARY COLUMN; 8 KV; 20 MM PHOSPHATE BUFFERE, PH 7.4. [(TM-AU-CN)$^-$] WAS CALCULATED FROM THE DIFFERENCE BETWEEN TOTAL GOLD CONCENTRATION AND MEASURED CONCENTRATIONS OF AUTM AND [AU(CN)$_2$]$^-$.

The equilibrium constant was calculated according to:

1/n $[AuTM]_n$ +[CN]$^-$ --$K_1$--> [TMAuCN]$^-$ (1)

[TMAuCN]$^-$ + [CN]$^-$ --$K_2$--> [Au(CN)$^2$]$^-$ + TM (2)

The overall equilibrium constant Kapp is defined as:

$K_{app} = K_1 * K_2 = ([Au(CN)_2]^- * [TM]/([AuTM] * [(CN)^-]_2) = 0.0006\ M^{-1}$ (3)

The equilibrium constant $K_2$ was most easily measured by studying the reverse reaction, that of thiomalic acid with dicyanogold(I). Figure 7 shows that reaction where the broad peak in the electropherogram occurring at ca. 21.5 min is clearly indicated to be the mixed ligand species [TMAuCN]$^-$. Details of the measurements and calculations are available in the dissertation of Z. Zhao (5).

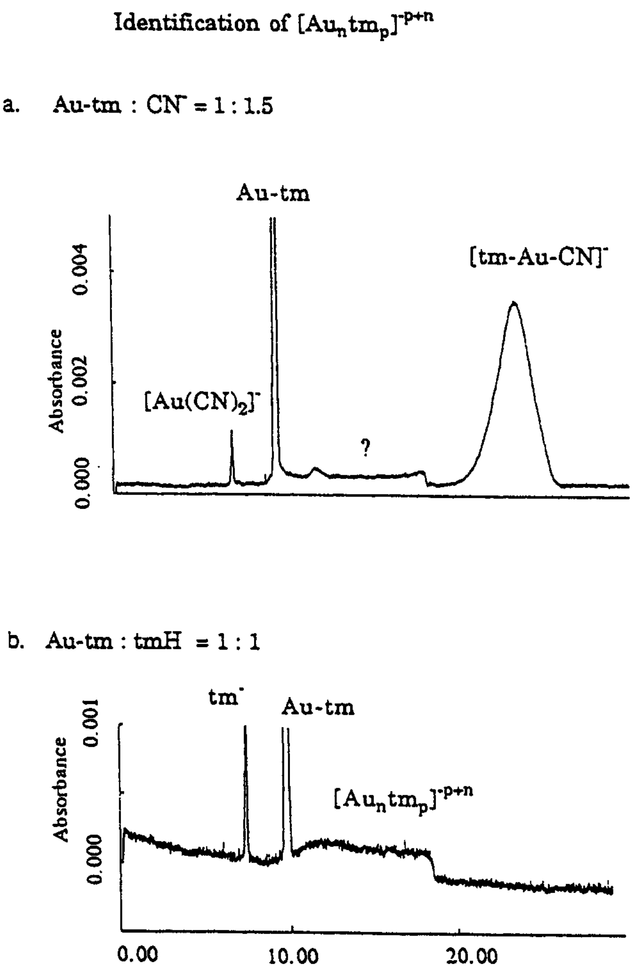

FIGURE 8 COMPARISON OF REACTIN OF (TOP) AUTM + KCN AND (BOTTOM) AUTM + TMH.

Dicyanogold(I), bis(cysteinato)gold(I) and cysteine calibration curves for absorbance vs. log concentration were calculated. A solution of dicyanogold(I), 1.0 mMg buffered to a pH of 7.4 with phosphate was titrated with cysteine and followed with CZE as shown in Figure 9. At a 1:1 ratio of dicyanogold(I) to cysteine four peaks were present in the electropherogram. Two peaks are attributed to cysteine and dicyanogold(I) and the two others are presumed to be bis(cysteinato)gold(I) and a mixed gold(I) species containing cyanide and cysteine. No dicyanogold(I) was left after a ratio of 1:100 dicyanogold(I) to cysteine was reached and only a trace amount of the mixed ligand species was present. The equilibrium constant was calculated as follows:

$$[Au(CN)_2]^- + cys \text{ --}K_1\text{--> } [cysAuCN]^- + CN^- \quad (4)$$

$$[cysAuCN]^- + cys \text{ --}K_2\text{--> } [Au\,(cys)_2]^- + CN^- \quad (5)$$

$$K_{eq} = K_1 * K_2([Au(cys)_2]^- * [CN^-]^2) / ([Au(CN)_2]^- * [cys]^2) = 0.006 \quad (6)$$

**Solution studies of $[Au(CN)_2]^-$ and N-acetylcysteine.** These were very similar in appearance to those with cysteine. Four peaks were apparent in the electropherogram. No equilibrium concentration measurements were attempted for this system.

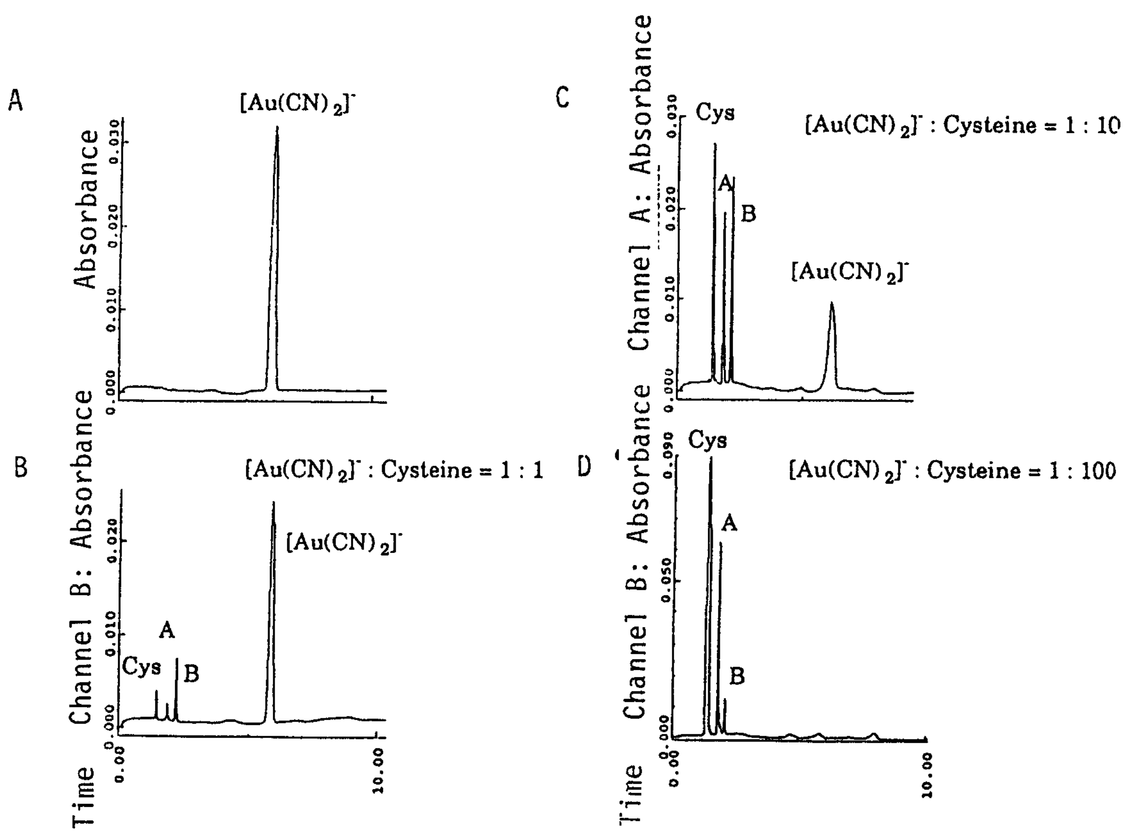

FIGURE 9 ELECTROPHEROGRAMS OF 1.0 MM $[Au(CN)_2]^-$ (A), MIXTURES OF $[Au(CN)_2]^-$ WITH CYSTEINE AT THE RATIOS OF $[Au(CN)_2]^-$/CYSTEINE = 1 (B), $[Au(CN)_2]^-$ /CYSTEINE = 1/10 (C) AND $[Au(CN)_2]^-$/CYSTEINE = 1/100 (D) IN 20 MM PHOSPHATE BUFFER, PH 7.4.

**Solution Studies of $[Au(CN)_2]^-$ and Glutathione.** Glutathione (GSH) is present in high concentrations inside cells and has a high binding affinity for gold(I). Because of the similarity to cysteine, we expected dicyanogold(I) to react with glutathione via a mixed ligand intermediate. Interestingly this is not observed. Reaction of dicyanogold(I) and GSH in 2:1 and 1:1 ratios showed that dicyanogold(I) is converted directly to $[Au(GSH)_2]$and no evidence for a mixed ligand species was present as can be seen in Figure 10. A product with the same migration time was also seen in the reaction of AuTM with GSH, ruling out the possibility that this peak is due to a $CN^-$ containing mixed ligand species. This failure to observe a mixed ligand species also is in agreement with the earlier $^{13}C$ NMR study of Lewis and Shaw (8). The pathway for this reaction has not yet been determined.

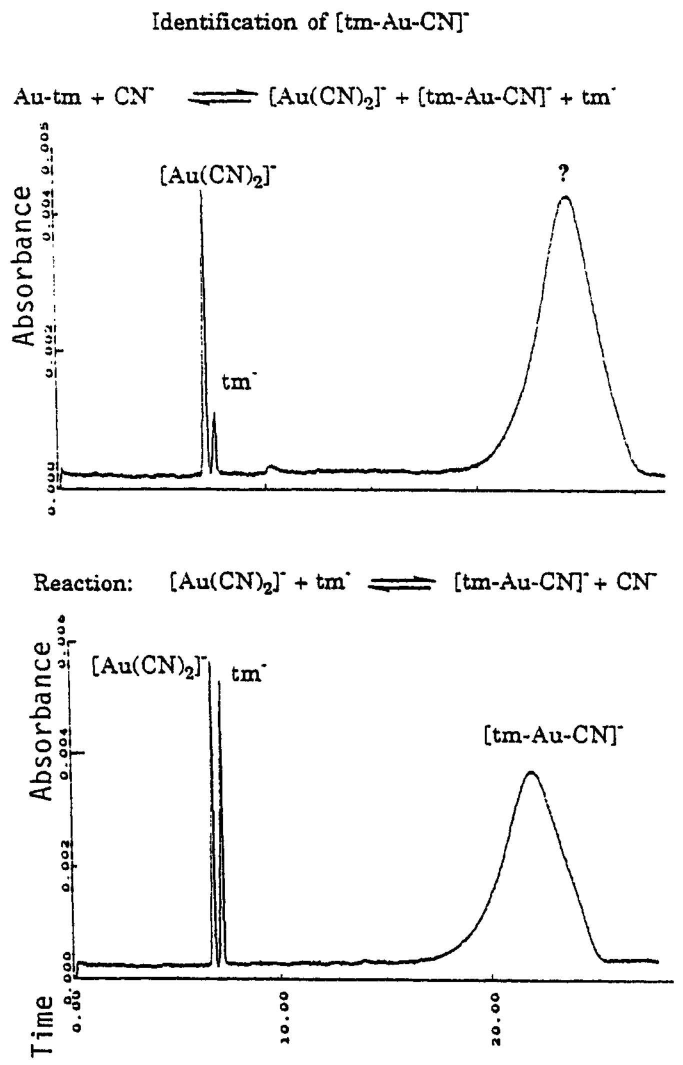

FIGURE 7 COMPARISON OF THE REACTION OF (TOP) AUTM + KCN AND (BOTTOM) $[Au(CN)_2]^-$ + tmH.

**Solution Studies of AuTM and Thiomalic Acid.** In order to observe the effect of added thiomalate on Myochrysine, solution studies in parallel to those earlier ones of Isab and Sadler (7) were undertaken using CZE. The electropherogram obtained is shown in Figure 8.

**Solution studies of $[Au(CN)_2]$ and Cysteine.** Biological fluids contain many species which bind gold(I) readily and compete with CN-. Thiol containing amino acids have a high affinity to bind gold(I) and are readily available in the blood stream. Two of the most abundant, cysteine (cys) and glutathione (GSH) were studied using CZE and the equilibrium constant for reaction of dicyanogold(I) and cysteine was calculated.

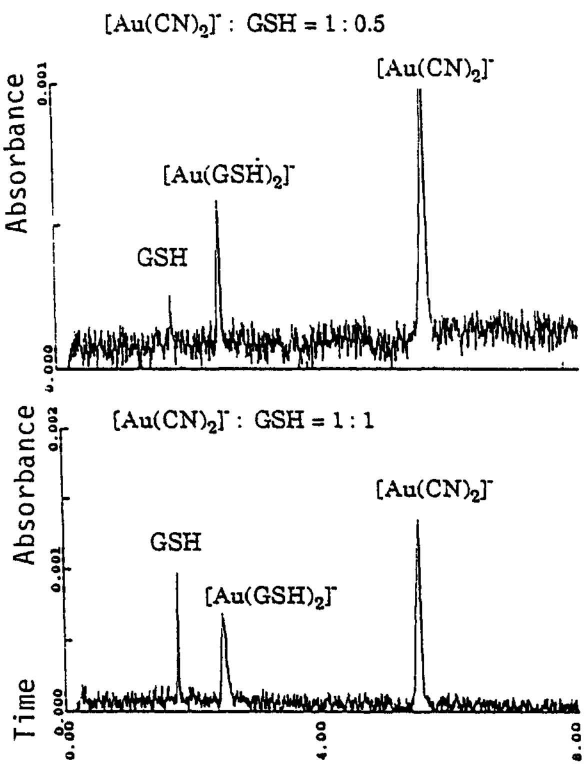

FIGURE 10 MIXTURE OF $[Au(CN)_2]^-$ AND GLUTATHIONE.

### Discussion

**Structure of Myochrysine in Solution.** The EXAFS experiments reported here show that the polymeric structure (previously discovered, using rather esoteric WAXS and DAS experiments) of AuTM (see Figure 1) is maintained at millimolar concentrations. The CZE experiments demonstrate that this same structure persists from millimolar to micromolar levels, such as those seen in the blood and urine of patients on chrysotherapy. Of course, these studies were performed in the absence of the additional endogenous thiols found in the human body, such as cys-34 of albumin, free cysteine or glutathione, which may break up the polymer in vivo. However, they do show that the AuTM (and presumably gold(I)thioglucose or Solganol) polymers are quite robust.

Additionally, these studies provide some information that the D- and L-forms of AuTM are no different from the racemic form used in chrysotherapy, in terms of their framework structure. Clearly more subtle differences with respect to in vivo interactions with chiral materials in the body are possible.

**Reactions of AuTM with Cyanide and Thiomalic acid.** Here CZE experiments with UV detection show that the reactions of KCN with AuTM produce a mixed ligand peak at ca. 21.5 min. Figures 5 and 7 (the latter produced via the reverse reaction of $[Au(CN)_2]^-$ with thiomalate) clearly demonstrate that this reaction produces a mixed ligand species corresponding to $[TMAuCN]^-$.

Interestingly, Figures 8a and 8b both show mixed oligomeric complexes of the type $[Au_nTM_p]^{p+n}$. It appears that the broad unresolved "blobs" which rise in both electropherograms originate from these polynuclear species in which the ratio of p to n is greater than 7 to 6. The lack of resolution in the separation of these materials suggests that they are a rapidly reequilibrating mixture with slightly different electropheretic behaviors for the individual components. The strikingly different behavior for "AuTM" (the sharp, well-defined peak at slightly less than 10 min) suggests a much greater stability for that particular species.

**Reactions of $[Au(CN)_2]^-$ with Thiols.** Clearly these reactions are not all of the same pattern. While cysteine and N-acetylcysteine require large excesses of thiol to force the reaction to completion and show evidence of a stable mixed ligand complex with one cyanide and one thiolate ligand, glutathione does not. The glutathione reaction requires less of an excess of the ligand and shows no evidence of a mixed ligand complex. Given the added steric bulk of this ligand compared to cysteine, it may be that some ligand-ligand interaction for bis(glutathione)gold(I) stabilizes the complex relative to other bis(thiolato)gold(I) complexes.

Finally, it is worth mention that, CZE is a powerful technique for the separation of metal complexes. Its utility for separation and characterization of metal-based drugs and metabolites has been demonstrated here.

**Acknowledgements**

The ICP-MS used in this work was purchased with Research Challenge funds awarded by the State of Ohio to the Biomedical Chemistry Research Center. Partial support came from NIH (GM35404) to RCE. ZZ and WBJ each thank the Department of Chemistry for graduate fellowships.

**References**

1. R.C. Elder, K. Ludwig, J.N. Cooper, M.K. Eidsness *J. Am. Chem. Soc.* **1985**, *107*, 5024. R.C. Elder, M.K. Eidsness *Chem. Rev.* **1987**, *87*, 1027.

2. S.R. Rudge, D. Perrett, A.J. Swannell, P.L. Drury, *J. Rheumatol.* **1984**, *11*, 150.

3. R.C. Elder, Z. Zhao, Y. Zhang, J.G. Dorsey, E.V. Hess, K. Tepperman *J. Rheumatol.* **1993**, *20*, 268.

4. M.K. Eidsness, Ph.D. Dissertation, University of Cincinnati, 1984.

5. Z. Zhao, Ph.D. Dissertation, University of Cincinnati, 1993.

6. G.G. Graham, J.R. Bales, M.R. Grootveld, P.J. Sadler *J. Inorg. Biochem.* **1985**, *25*, 163.

7. A.A. Isab, P.J. Sadler *J. Chem. Soc. Dalton Trans.* **1982,** 135.

8. G. Lewis, C.F. Shaw *Inorg. Chem.* **1986**, *25*, 58.

